# New Endohyphal Relationships between Mucoromycota and *Burkholderiaceae* Representatives

**DOI:** 10.1128/AEM.02707-20

**Published:** 2021-03-11

**Authors:** Alicja Okrasińska, Aleksandra Bokus, Katarzyna Duk, Aleksandra Gęsiorska, Blanka Sokołowska, Aleksandra Miłobędzka, Marta Wrzosek, Julia Pawłowska

**Affiliations:** a Institute of Evolutionary Biology, Faculty of Biology, University of Warsaw, Warsaw, Poland; b Department of Water Technology and Environmental Engineering, University of Chemistry and Technology Prague, Prague, Czech Republic; c Botanic Garden, Faculty of Biology, University of Warsaw, Warsaw, Poland; University of Tartu

**Keywords:** BRE, *Mortierella*, *Umbelopsis*, bacterial-fungal interactions, endosymbionts

## Abstract

Mucoromycota representatives are known to harbor two types of endohyphal bacteria (EHB)—*Burkholderia*-related endobacteria (BRE) and *Mycoplasma*-related endobacteria (MRE). While both BRE and MRE occur in fungi representing all subphyla of Mucoromycota, their distribution is not well studied. Therefore, it is difficult to resolve the evolutionary history of these associations in favor of one of the following two alternative hypotheses explaining their origin: “early invasion” and “late invasion.” Our main goal was to fill this knowledge gap by surveying Mucoromycota fungi for the presence of EHB. We screened 196 fungal strains from 16 genera using a PCR-based approach to detect bacterial 16S rRNA genes, complemented with fluorescence *in situ* hybridization (FISH) imaging to confirm the presence of bacteria within the hyphae. We detected *Burkholderiaceae* in ca. 20% of fungal strains. Some of these bacteria clustered phylogenetically with previously described BRE clades, whereas others grouped with free-living *Paraburkholderia*. Importantly, the latter were detected in Umbelopsidales, which previously were not known to harbor endobacteria. Our results suggest that this group of EHB is recruited from the environment, supporting the late invasion scenario. This pattern complements the early invasion scenario apparent in the BRE clade of EHB.

**IMPORTANCE** Bacteria living within fungal hyphae present an example of one of the most intimate relationships between fungi and bacteria. Even though there are several well-described examples of such partnerships, their prevalence within the fungal kingdom remains unknown. Our study focused on early divergent terrestrial fungi in the phylum Mucoromycota. We found that ca. 20% of the strains tested harbored bacteria from the family *Burkholderiaceae*. Not only did we confirm the presence of bacteria from previously described endosymbiont clades, we also identified a new group of endohyphal *Burkholderiaceae* representing the genus *Paraburkholderia*. We established that more than half of the screened *Umbelopsis* strains were positive for bacteria from this new group. We also determined that, while previously described BRE codiverged with their fungal hosts, *Paraburkholderia* symbionts did not.

## Abstract (precis)

Bacteria living within fungal hyphae present an example of one of the most intimate relationships between fungi and bacteria. Even though there are several well-described examples of such partnerships, their prevalence within the fungal kingdom remains unknown.

## INTRODUCTION

Interactions between fungi and bacteria are common and widespread in the environment, as these organisms often occupy similar niches, and together, they are responsible for most decomposition processes in soil (1). However, not only can bacteria live in close proximity with the fungus, sometimes they are also present inside the fungal hyphae. Bacteria occupying this specific niche are referred to as endohyphal bacteria (EHB). EHB were first observed in spores of *Endogone* by Mosse in 1970 (2), although they were then described as “bacteria-like structures.”

Despite observing these bacteria-like structures in *Endogone*, the first formally described endobacterium associated with the Mucoromycota representative was “*Candidatus* Glomeribacter gigasporarum,” which was found to be living inside the hyphae of Gigasporales (3), and its prevalence seems to be limited to this order. “*Candidatus* Glomeribacter gigasporarum” belongs to *Burkholderiaceae* family and is considered one of the *Burkholderia*-related endobacteria (BRE). These bacteria are known to be vertically transmitted and were detected in both mycelium and spores (3). They help the fungus form a relationship with the plant by increasing ATP production and inducing detoxification of reactive oxygen forms (4). Another similar partnership involves Rhizopus microsporus (Mucoromycotina) and at least two species of BRE (*Mycetohabitans rhizoxinica* and *Mycetohabitans endofungorum* [Paraburkholderia rhizoxinica {synonym}] and Paraburkholderia endofungorum) (5, 6). In this relationship, bacteria were demonstrated to completely control asexual reproduction as well as partially control sexual reproduction of their host (7). More recently, a partnership between *Mortierella elongata* (Mortierellomycotina) and Mycoavidus cysteinexigens was discovered and described (8, 9). This partnership has been studied extensively, with both partners having their genomes assembled and annotated and series of physiological experiments carried out. It has been demonstrated that the bacterium relies on the fungal cysteine and that the host growth rate is higher in cured strains (9). There are also reports of endohyphal bacteria in Dikarya (10). In this group, single fungal hosts were shown to harbor few different bacterial endosymbionts from evolutionarily distant lineages (e.g., reference 11), which is not the case of Mucoromycota. Fungi from this phylum usually harbor one or two different lineages of bacteria, and these are quite uniform across the whole phylum, suggesting their evolutionary ancient relationship.

All of the above-mentioned interactions involving Mucoromycota representatives also involve bacteria from the *Burkholderiaceae* family (*Burkholderiales*, *Betaproteobacteria*, *Proteobacteria*), which are Gram-negative rod-shaped bacteria. Representatives of this clade are omnipresent in the environment, colonizing a wide range of ecological niches, from soil ecosystems to the human body; this family consists of obligately aerobic, facultatively anaerobic chemoorganotrophs as well as chemolithotrophs, both obligate and facultative (12). Recent phylogenomic studies of *Burkholderia sensu lato* led to the division of this genus into several new genera that seem to reflect the most prevalent lifestyle within the genus as well as phylogenetic grouping (13). The genus *Paraburkholderia* comprises environmental strains known to be sometimes beneficial to plants and *Burkholderia sensu stricto* comprises human and animal pathogens, while the newly established genus *Mycetohabitans* comprises two BRE isolated from Rhizopus microsporus (*Mycetohabitans rhizoxinica* and *Mycetohabitans endofungorum*) (14).

Another lineage of bacteria detected in the representatives of Glomeromycotina as well as in the *Mortierella* genus and Endogonales order is *Mycoplasma*-related (*Mycoplasmataceae*, *Mollicutes*, *Tenericutes*) endobacteria (MRE). Nauman et al. (15) identified MRE in Glomeromycotina, which was later confirmed by Naito et al. (16). Then, Desirò et al. (17) found MRE in *Mortierella* strains and concluded, after curing fungi of their endosymbionts, that these bacteria seem to be mild parasites. MRE were also detected in three out of four recently studied genomes of Endogonales (Mucoromycotina) representatives (18). Apart from being endosymbionts, bacteria from the family *Mycoplasmataceae* can lead a saprotrophic or parasitic lifestyle as well.

Together, these findings indicate that interactions between Mucoromycota fungi and bacteria are common and that they have been neglected for years (or there were no methods of studying them). Pawlowska et al. (19) suggested that it is inevitable to find new Mucoromycota-endosymbiont partnerships, and thus, looking for endosymbiotic bacteria as a part of primary research for each new species is recommended. In the same year, Takashima et al. (20) published a study in which they screened 238 strains of environmental *Mortierella* isolates originating from Japan using PCR and fluorescence *in situ* hybridization (FISH). They report that about 20% of the strains harbored BRE, which can be separated into three new subclades (called A, B, and C), but the authors were not able to draw conclusions about the factors driving these interactions. Moreover, they performed a FISH procedure confirming location of BRE inside the hyphae for five isolates. MRE seem to be less common within Mortierellomycotina, as Desirò et al. (17) report that only 12 out of 394 strains (ca. 3%) possess the bacteria in question.

Clearly, studying Mucoromycota-bacterial relationships can also help our understanding of the evolutionary history of interactions between fungi and bacteria. Mucoromycota are commonly described as early diverging, as they are one of the most ancient groups of land fungi (21). The Mucoromycota phylum comprises three subphyla: Glomeromycotina, Mortierellomycotina, and Mucoromycotina (22). The first one is rather uniform in the trophic mode of its representatives—almost all Glomeromycotina fungi are obligate endomycorrhizal partners of plants (with the exception of *Geosiphon pyriformis*, which forms a relationship with endosymbiotic cyanobacteria) (23). Mortierellomycotina are common and ubiquitous soil saprotrophs with a worldwide distribution (24), and are thought to form nonobligatory relationships with plant roots (25–27). Mucoromycotina is the most diverse subphylum in the phylum and encompasses the following three clearly distinct orders: Endogonales, Mucorales, and Umbelopsidales. Endogonales are mainly obligatory plant symbionts (28), while representatives of the other two orders are mostly common soil saprotrophs. However, many representatives of Mucorales are also isolated from spoiled fruits, vegetables, mushrooms, or bread (e.g., *Rhizopus* spp., *Mucor* spp., *Choanephora cucurbitarum*). There are also rare examples of Mucorales acting as opportunistic pathogens in immunocompromised patients (causing mucormycosis) (29).

The patterns of presence and absence of BRE and MRE in Mucoromycota representatives enabled Bonfante and Desirò (30) to propose the following two different hypotheses on the evolution of bacterial-fungal interactions: early and late bacterial invasion. While these hypotheses apply to both types of EHB, in this paper, we focus on BRE. The early bacterial invasion hypothesis states that the common ancestor of all present BRE interacted with the common ancestor of extant Mucoromycota representatives. The diversity of BRE that we can observe today is thus a result of a codiversification of hosts and endosymbionts. The late bacterial invasion hypothesis states that there is at least some level of horizontal acquisition of BRE by representatives of different Mucoromycota lineages, which can also explain present day diversity of BRE. However, both scenarios are based on scarce data, especially considering the lack of information about EHB in the representatives of Mucoromycotina other than Endogonales and *Rhizopus*.

Therefore, the main goal of our study was to screen chosen fungal representatives of the Mucoromycota phylum for the presence of endohyphal bacteria and identify potential coevolution patterns in order to support or modify the current hypotheses on the evolution of bacterial-fungal partnerships within Mucoromycota. We included representatives of genera and orders underrepresented in endobacterial studies, such as *Umbelopsis*.

## RESULTS

Among 196 strains belonging to 16 genera within the Mucoromycota phylum, 42 were demonstrated to be positive for bacteria from the *Burkholderiaceae* family, which constitutes 21% of all screened isolates (Table 1). As expected and previously reported (20), nearly 20% of *Mortierella* strains harbored bacteria from *Burkholderiaceae*. We also observed interactions between *Umbelopsis* and *Burkholderiaceae*. More than half (23 of 40) of screened strains of this genus tested positive for *Burkholderiaceae*. There was one strain (out of 15) of *Mucor* which seemed to have a relationship with *Burkholderiaceae* bacteria as well. As was expected, none of the screened arbuscular mycorrhizal fungi tested positive for the presence of bacteria from this group. However, we also identified *Mycoplasma*-related bacteria in *Mortierella formicae*, *Diversispora* sp., and two species of *Glomus* (data not shown). Some of the strains have had PCR bacterial product, but the identified bacteria belong to neither of the described groups. All of the data can be found in Table 1.

**TABLE 1:** Prevalence of *Burkholderiaceae* in each screened genus^*a*^

Subphylum	Order	Family	Genus	No. of strains tested	No. of strains with *Burkholderiaceae*
Glomeromycotina	Diversisporales	Diversisporaceae	*Diversispora*	2	0
Glomeromycotina	Glomerales	Glomeraceae	*Glomus*	5	0
Glomeromycotina	Glomerales	Claroideoglomeraceae	*Claroideoglomus*	1	0
Mortierellomycotina	Mortierellales	Mortierellaceae	*Mortierella*	76	15
Mucoromycotina	Mucorales	Chaetocladiaceae	*Chaetocladium*	1	0
Mucoromycotina	Mucorales	Cunninghamellaceae	*Absidia*	2	0
Mucoromycotina	Mucorales	Cunninghamellaceae	*Cunninghamella*	3	0
Mucoromycotina	Mucorales	Gilbertellaceae	*Gilbertella*	1	0
Mucoromycotina	Mucorales	Lichtheimiaceae	*Lichtheimia*	2	0
Mucoromycotina	Mucorales	Mucoraceae	*Actinomucor*	3	0
Mucoromycotina	Mucorales	Mucoraceae	*Mucor*	15	1
Mucoromycotina	Mucorales	Mucoraceae	*Rhizopus*	42	3
Mucoromycotina	Mucorales	Syncephalastraceae	*Syncephalastrum*	1	0
Mucoromycotina	Mucorales	Thamnidiaceae	*Thamnidium*	1	0
Mucoromycotina	Calcarisporiellales	Calcarisporiellaceae	*Calcarisporiella*	1	0
Mucoromycotina	Umbelopsidales	Umbelopsidaceae	*Umbelopsis*	40	23
Total				196	42

a The systematic classification follows the backbone proposed by Spatafora et al. (22).

**FIG 1:**
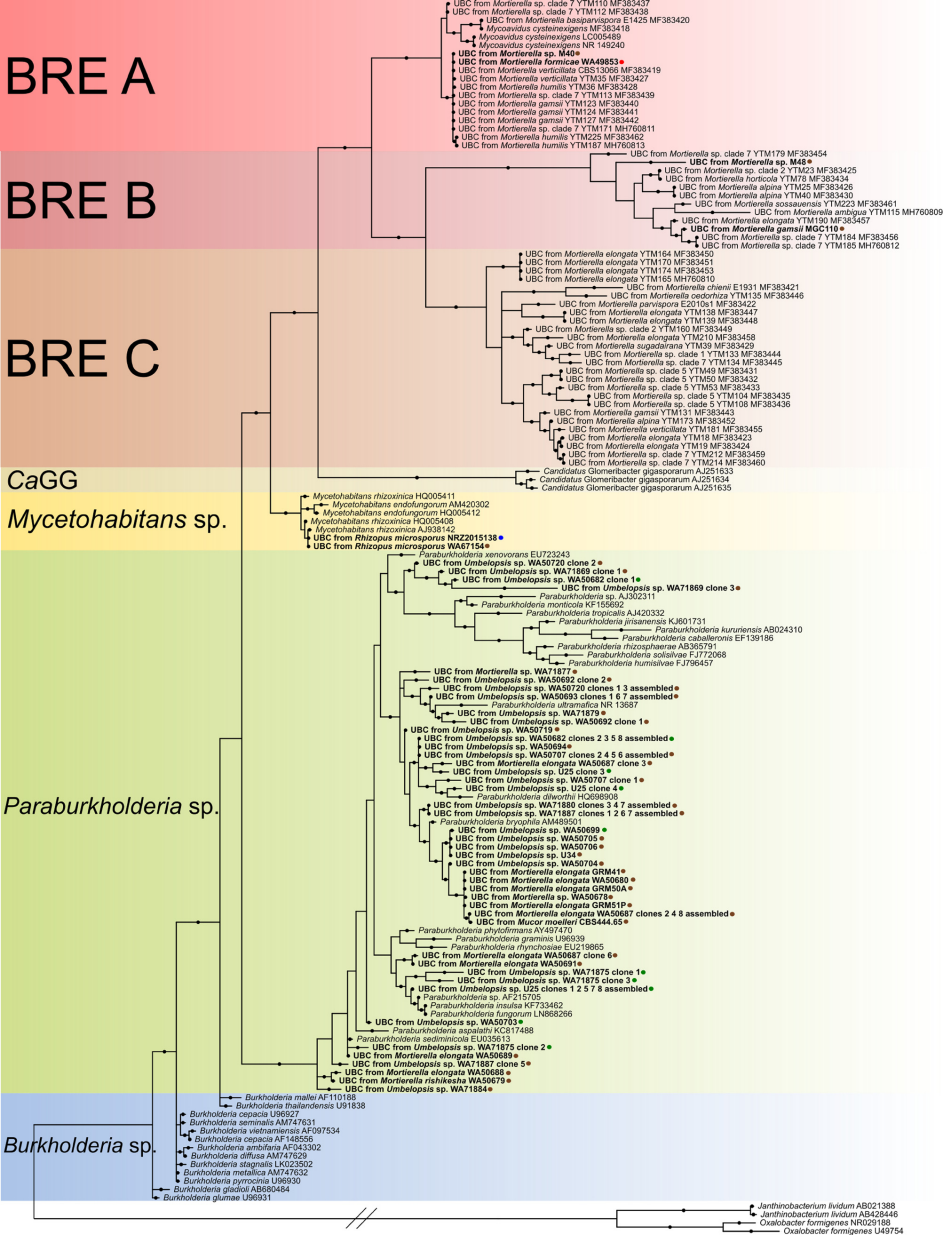
Bayesian phylogenetic tree of *Burkholderiaceae*-related endosymbionts based on partial 16S rRNA genes calculated as described in Materials and Methods. Branches marked with dots have posterior Bayesian probability higher than 0.8. Colors in the background indicate groups of sequences (from the top, three red clades of *Burkholderia*-related endosymbionts, A, B, and C; orange, “*Candidatus* Glomeribacter gigasporarum”; yellow, *Mycetohabitans* sp.; green, *Paraburkholderia* sp.; blue, *Burkholderia* sp.). UBC is an abbreviation for uncultured bacterial clone. Tips in bold are the ones obtained during this study. Colors of the dots next to the names indicate the source from which the hosts were obtained as follows: brown, soil; green, plant; blue, human with mucormycosis; and red, an ant. Two 16S rRNA gene sequences of Oxalobacter formigenes and two 16S rRNA gene sequences of Janthinobacterium lividum were used as an outgroup.

After selection of bacteria belonging to the *Burkholderiaceae* family, we reconstructed the phylogeny using 16S rRNA gene sequences of previously found *Burkholderia*-related endobacteria as well as free-living *Burkholderiaceae*. Our analysis showed that the identified *Burkholderiaceae* do not form a uniform group (Fig. 1). All obtained sequences split into the following two groups within *Burkholderiaceae*: one comprised strictly of endohyphal strains from Rhizopus microsporus, *Mortierella* spp., and arbuscular mycorrhizal fungi (Mycoavidus cysteinexigens [clades A, B, and C], *M. rhizoxinica/M. endofungorum*, and “*Candidatus* Glomeribacter gigasporarum” in Fig. 1; hereinafter referred to as BRE); and the other consisting mainly of free-living, environmental *Paraburkholderia* strains and endohyphal clones from this study (highlighted in green in Fig. 1). In the BRE clade, three main lineages can be distinguished, corresponding to *Mycetohabitans* spp., “*Candidatus* Glomeribacter gigasporarum,” and Mycoavidus cysteinexigens. Takashima et al. (20) further divided the *Mycoavidus* clade into three subclades, A, B, and C, and this pattern is also visible in our analysis. Two out of 15 endohyphal clones from our *Mortierella* spp. grouped within subclade A, and the same number grouped within subclade B (Fig. 1). None of our *Burkholderiaceae* sequences grouped within subclade C. Sequences obtained from Rhizopus microsporus all grouped within the *Mycetohabitans* spp. clade. The remaining 11 sequences of *Burkholderiaceae* from *Mortierella* strains, as well as all sequences from *Umbelopsis* spp. and one from *Mucor moelleri*, grouped with environmental sequences of *Paraburkholderia sensu stricto* (highlighted in green in Fig. 1).

**FIG 2:**
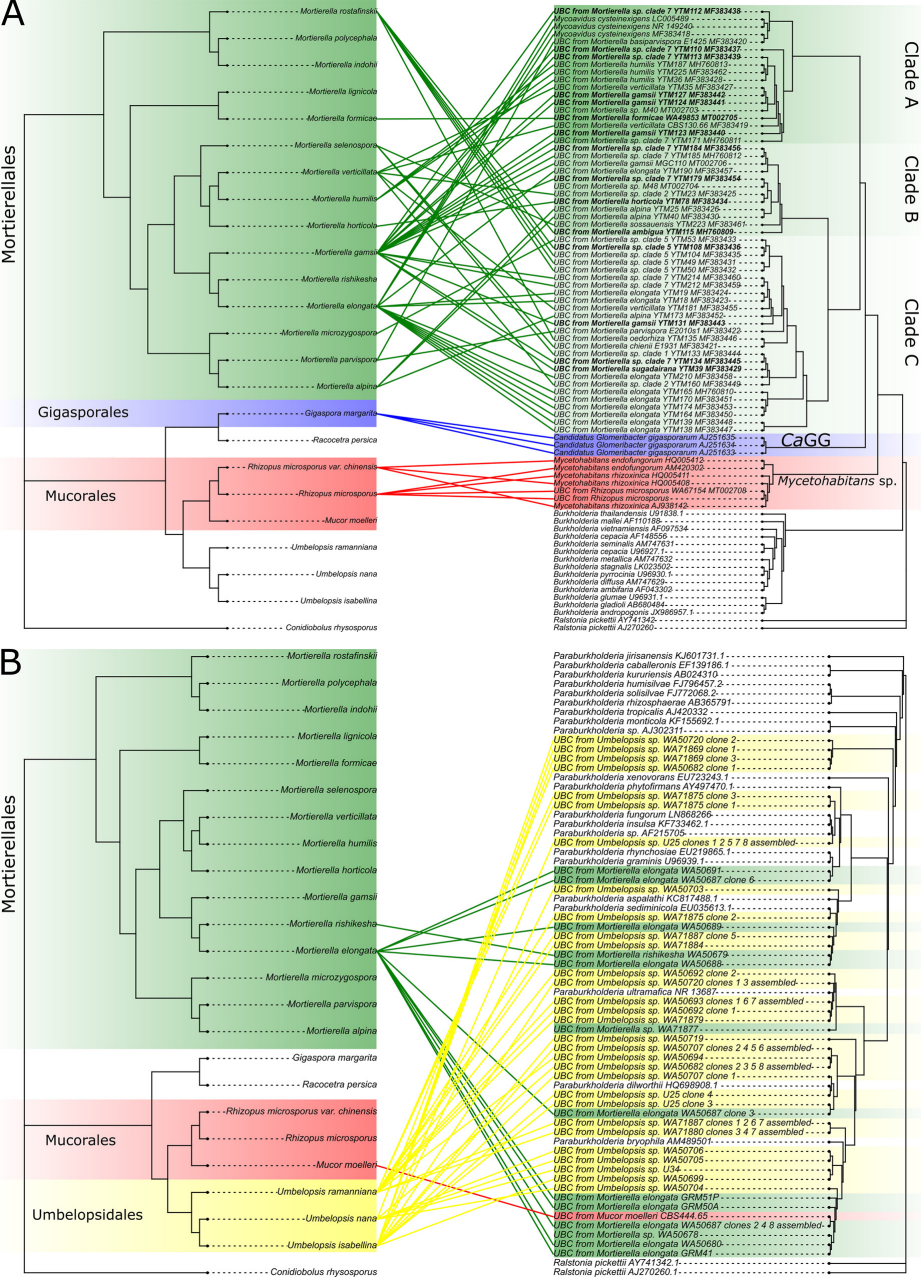
Tanglegrams of cophylogenetic relationships between fungal hosts (left) and their bacterial endosymbionts (right, BRE [A] and PRE [B]). All three trees were calculated using RAxML-NG as described in Materials and Methods. Color of highlight and links denote fungal orders and bacteria associated with its representatives. The links that were found to be statistically significant by ParaFit are denoted by bolding the bacterial tips. If the exact phylogenetic placement of the host was not known, the link was drawn to the closest species.

ParaFit analyses of bacteria and fungus phylogenies indicated an overall pattern of cospeciation in the BRE clade (ParaFitGlobal, *P* = 0.004), whereas it was not observed in the *Paraburkholderia* group (ParaFitGlobal, *P* = 1). Individual links tested with ParaFit can be found in Fig. 2.

**FIG 3:**
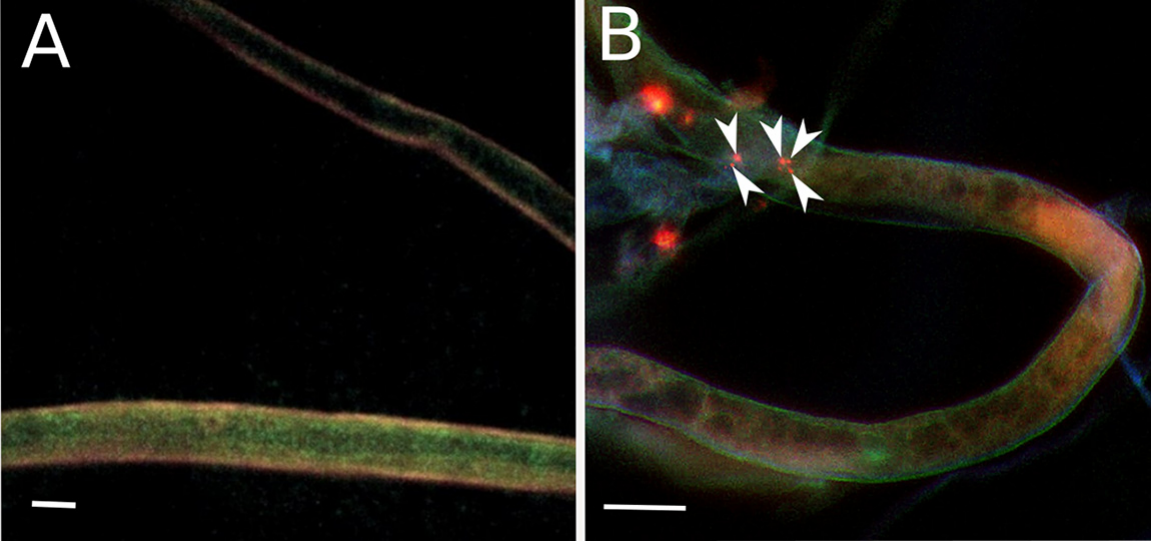
FISH visualization of endohyphal bacteria. Negative control (A) and in *Umbelopsis* sp. WA50699 (Umbelopsidales, Mucoromycotina) (B); bacterial cells within hyphae are indicated by arrowheads. Bar = 10 μm.

FISH confirmed the presence of endohyphal bacteria inside the living mycelia of Rhizopus microsporus (picture not shown), *Umbelopsis* sp. (Fig. 3B), and *Mortierella elongata* (picture not shown). For *Mortierella elongata* WA50687, three-dimensional visualization of hyphae with endohyphal bacteria was prepared (31). Although the identity of detected EHB was not confirmed by species-specific probe, it is highly likely that visualized bacteria are the ones detected by PCR, as usually only one strain of bacteria was identified in one fungal strain. Even in EHB-positive strains, bacterial cells were not present in all hyphae; for each strain, multiple empty hyphae were also observed (Fig. 3A).

## DISCUSSION

In this study, we aimed to expand knowledge about endohyphal bacteria living within hyphae of Mucoromycota representatives. To achieve this goal, we decided to use molecular methods for screening fungal isolates for the presence of bacteria and fluorescence microscopy for the confirmation of the endohyphal nature of the detected bacteria.

Currently, representatives of Mucoromycota are known to harbor endosymbionts from two distinct bacterial lineages—*Burkholderia*- and *Mycoplasma*-related ones (30)—in their hyphae, with the former probably being more widespread than the latter. In our search for EHB from previously undersampled lineages of this phylum, we detected bacteria that were previously undescribed. However, we also detected numerous endobacteria from already established clades of BRE and report similar percentage (20%) of BRE-harboring *Mortierella* as in the study conducted by Takashima et al. (20). All previously known lineages of BRE, including “*Candidatus* Glomeribacter gigasporarum” and Mycoavidus cysteinexigens, as well as a portion of BRE detected in *Mortierella* during this study, form a sister clade to *Mycetohabitans*. Notably, all endosymbionts of *Umbelopsis* spp. detected in our study grouped with environmental *Paraburkholderia* strains, and the endohyphal nature of this relationship is postulated.

At first glance, the prevalence of EHB in different lineages of Mucoromycota does not seem to be correlated with the phylogenetic position of the fungal host. Even though on a subphylum level, the colonization frequency is similar in Mortierellomycotina and Mucoromycotina (ca. 20% and ca. 24%, respectively); on an order level, the highest prevalence of EHB was observed in the representatives of Umbelopsidales (ca. 58%). The highest percentage of BRE-positive strains was observed among the strains isolated from soil (ca. 31%), and the lowest (apart from substrates represented by less than 5 strains) from clinical strains (ca. 4%). We want to elucidate, however, that the number of strains in our study does not allow us to draw conclusions about the impact of ecological niche on the prevalence of EHB and that the influence of fungal isolation substrate on prevalence and identity of EHB within Mucoromycota should be further investigated.

*Paraburkholderia* spp. seem to be plant associated (usually being isolated from rhizosphere), and some strains can potentially have a positive impact on plant health (13, 14). As more than one-half of the screened strains of *Umbelopsis* spp. tested positive for *Paraburkholderia* sp. and one-third of them were isolated from the plant material, we hypothesize that the relationship between fungus and bacteria could be beneficial for plants, especially since it is postulated that the fungal role in relationships with endohyphal bacteria is providing a safe environment for them. However, data are still scarce, and physiological experiments using cleared and infected isogenic isolates, similar to those performed by Uehling et al. (9), as well as comparative transcriptomics experiments of such strains (32) and sampling more strains from different locations around the world, are needed to assess the actual impact of *Umbelopsis* on plants as well as to examine how this impact changes with the presence of endobacteria.

The ancient origin (350 to 400 million years ago [MYA]) of BRE (*Mycoavidus-Glomeribacter-Mycetohabitans* lineage) in Mucoromycota was postulated by Mondo et al. (7) and Uehling et al. (9). Their results would thus support the early BRE invasion hypothesis in Mucoromycota as proposed by Bonfante and Desirò (30). In our study, the coevolution of EHB and fungal hosts may be observed on the order level in the BRE clade (i.e., Mortierellales, *Mycoavidus sensu lato*; Gigasporales, “*Candidatus* Glomeribacter sp.”; Mucorales, *Mycetohabitans* spp.). We also prove that there is a significant coevolutionary pattern between Mucoromycota and BRE *sensu stricto*, which suggests that the common ancestor of this clade interacted with an ancestor of Mucoromycota and they coevolved from this moment onwards. Conversely, our results support the late invasion hypothesis for bacteria identified as *Paraburkholderia* spp. It seems that symbionts from this genus may be recruited from the environment when it is advantageous for partners and form more transient relationships with Mucoromycota than BRE. Our hypothesis is in agreement with the final conclusion of Bonfante and Desirò (30). They hypothesize that soil, with its living components, has acted as a facilitator in transferring free-living bacteria inside fungi. We also speculate that the event of interaction between ancestors of Mucoromycota and BRE may have enabled fungi to interact with different types of bacteria. That would explain why *Paraburkholderia* representatives, closely related to BRE, were found in closely related *Mortierella* and *Umbelopsis*. As the current state of knowledge is largely incomplete, further studies are required to fully understand the nature of initiating and maintaining relationships between fungi and bacteria as well as their evolutionary origin.

In conclusion, screening of 196 fungal strains of Mucoromycota revealed EHB from the *Burkholderiaceae* family in ca. 20% of them. Some of the detected bacteria could be assigned to previously described endosymbiotic clades (*Mycoavidus sensu lato*, *Mycetohabitans* spp.), but others clustered with free-living *Paraburkholderia*. Most importantly, this study allowed for identification of potentially endohyphal bacteria in *Umbelopsis* spp. belonging to *Paraburkholderia* spp. The hypotheses regarding the time of invasion of EHB in Mucoromycota could not be resolved with certainty. However, we lean toward the early invasion hypothesis for BRE and the late invasion hypothesis for *Paraburkholderia* spp.

## MATERIALS AND METHODS

### Fungal strains collection and identification.

**FIG 4:**
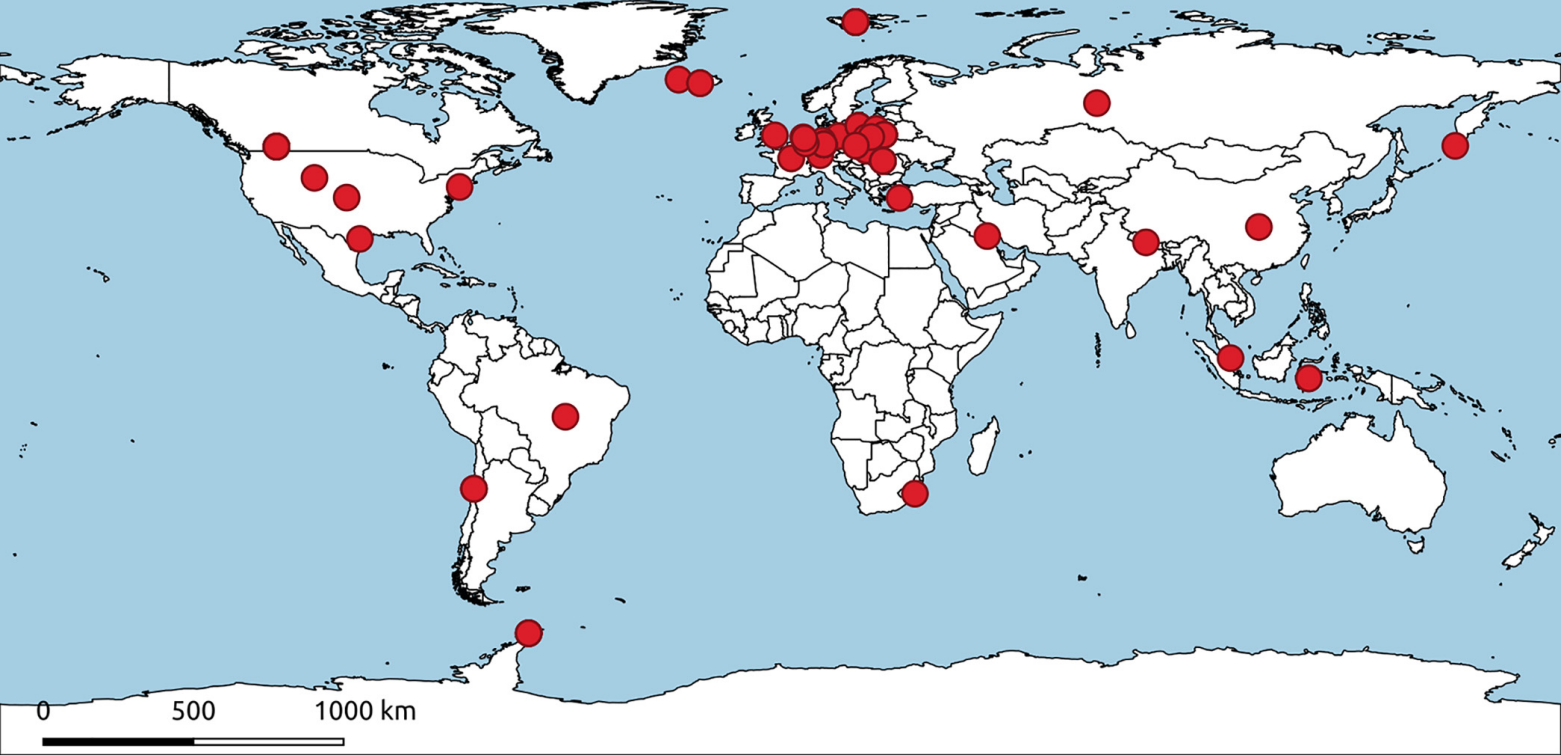
World map showing locations from which fungal strains were obtained (projection EPSG:4326-WGS84). Each red dot represents one strain. If the exact isolation location is known (location name or coordinates), the dot is placed as accurately as possible. If not, the dot is placed in the geographic center of the country.

Between 2015 and 2019, we collected soil from Europe, Antarctica, and the Arctic. Mucoromycotina and Mortierellomycotina representatives were isolated from soil using the Warcup method on water agar (WA) plates (33). Emerging hyphae were subsequently transferred to new malt extract agar (MEA) plates in order to obtain pure colonies of each strain. Since culturing obligate biotrophs is difficult, Glomeromycotina spores were suspended in water and used for further analysis. Additionally, 32 strains from the Westerdijk Fungal Biodiversity Institute culture collection, 39 strains from the Nationales Referenzzentrum (NRZ) für Mykobakterien culture collection, and 8 strains from the Jagiellonian University collection were also used in this study. A detailed list of all of the strains used and sampling sites is presented in Table 2 and Table S1 in the supplemental material and is visualized in Fig. 4. The map of sampling sites was prepared using qGIS 3.4 Madeira (34).

**TABLE 2:** Fungal strains used in the study

Strain ID	Phylogenetic placement	Place of origin
Act	*Actinomucor elegans*	Poland
AG	*Absidia glauca*	Poland
BEG11	*Glomus geosporum*	Poland
BEG12	*Glomus mosseae*	Poland
BEG144	*Glomus intraradices*	Poland
BEG23	*Glomus claroideum*	Poland
CBS 101040	Lichtheimia corymbifera	France
CBS 102.35	*Absidia fusca*	Germany
CBS 103.35	Lichtheimia ramosa	ND^*a*^
CBS 117697	*Actinomucor kuwaitensis*	Kuwait
CBS 120585	*Mucor indicus*	France
CBS 120811	*Syncephalastrum racemosum*	ND
CBS 123972	*Mucor hiemalis*	Germany
CBS 123973	Mucor circinelloides	Germany
CBS 142.35	*Mucor circinatus*	Brazil
CBS 156.74	*Chaetocladium brefeldii*	The Netherlands
CBS 167.53	*Cunninghamella elegans*	Canada
CBS 185.68	*Mucor jensenii*	Russia
CBS 185.77	*Mucor amphibiorum*	USA
CBS 190.32	*Gilbertella persicaria*	USA
CBS 222.81	*Mucor racemosus*	The Netherlands
CBS 226.32	*Mucor plumbeus*	Canada
CBS 236.35	*Mucor lusitanicus*	Germany
CBS 242.35	*Mucor hiemalis*	Germany
CBS 243.67	*Mucor jensenii*	South Africa
CBS 308.87	Rhizopus microsporus	USA
CBS 318.78	*Cunninghamella elegans*	Turkey
CBS 338.72	*Actinomucor elegans*	Nepal
CBS 366.70	Mucor circinelloides	The Netherlands
CBS 372.95	*Cunninghamella bertholletiae*	China
CBS 411.52	*Thamnidium elegans*	Poland
CBS 422.71	*Mucor indicus*	Indonesia
CBS 444.65	*Mucor moelleri*	USA
CBS 515.94	Rhizopus arrhizus	Singapore
CBS 969.68	Mucor circinelloides	Russia
CBS279.70	*Calcarisporiella thermophila*	England
H2C1	*Mortierella elongata*	Iceland
H2C2	*Mortierella elongata*	Iceland
J6	*Glomus claroideum*	Poland
M21	*Mortierella* sp.	Canada
M19	*Mortierella* sp.	Canada
M23	*Mortierella* sp.	Canada
M25	*Mortierella alpina*	Romania
M26	*Mortierella* sp.	Canada
M4	*Mortierella parvispora*	Poland
M40	*Mortierella* sp.	Canada
M44	*Mortierella* sp.	Canada
M48	*Mortierella* sp.	Canada
M54	*Mortierella gamsii*	Romania
MGC110	*Mortierella gamsii*	Poland
MGC142	*Mortierella zychae*	Poland
N1131	*Mortierella alpina*	The Arctic
N1231A	*Mortierella minutissima*	The Arctic
N1525	*Mortierella hyalina*	The Arctic
N2121	*Mortierella minutissima*	The Arctic
N2131	*Mortierella hyalina*	The Arctic
N3532	*Mortierella minutissima*	The Arctic
N4235	*Mortierella minutissima*	The Arctic
N4323B	*Mortierella gamsii*	The Arctic
N4332	*Mortierella minutissima*	The Arctic
N4421	*Mortierella minutissima*	The Arctic
N4422	*Mortierella minutissima*	The Arctic
N5321	*Mortierella alpina*	The Arctic
N5431B	*Mortierella alpina*	The Arctic
N5531	*Mortierella alpina*	The Arctic
N6431	*Mortierella alpina*	The Arctic
NRZ-2015-138	Rhizopus microsporus	Germany
NRZ-2015-182	Rhizopus arrhizus	Germany
NRZ-2015-216	Rhizopus microsporus	Germany
NRZ-2016-056	Rhizopus arrhizus	Germany
NRZ-2016-117	Rhizopus microsporus	Germany
NRZ-2016-214	Rhizopus microsporus	Germany
NRZ-2016-230	Rhizopus arrhizus	Germany
NRZ-2016-254	Rhizopus arrhizus	Germany
NRZ-2016-325	Rhizopus arrhizus	Germany
NRZ-2016-328	Rhizopus arrhizus	Germany
NRZ-2017-035	Rhizopus microsporus	Germany
NRZ-2017-167	Rhizopus microsporus	Germany
NRZ-2017-218	Rhizopus microsporus	Germany
NRZ-2017-239	Rhizopus microsporus	Germany
NRZ-2017-267	Rhizopus microsporus	Germany
NRZ-2017-370	Rhizopus microsporus	Germany
NRZ-2017-401	Rhizopus arrhizus	Germany
NRZ-2017-426	Rhizopus arrhizus	Germany
NRZ-2017-431	Rhizopus arrhizus	Germany
NRZ-2018-015	Rhizopus microsporus	Germany
NRZ-2018-028	Rhizopus microsporus	Germany
NRZ-2018-083	Rhizopus arrhizus	Germany
NRZ-2018-084	Rhizopus arrhizus	Germany
NRZ-2018-111	Rhizopus arrhizus	Germany
NRZ-2018-169	Rhizopus microsporus	Germany
NRZ-2018-178	Rhizopus microsporus	Germany
NRZ-2018-330	*Rhizopus stolonifer*	Germany
NRZ-2018-357	Rhizopus microsporus	Germany
NRZ-2018-385	Rhizopus arrhizus	Germany
NRZ-2018-414	Rhizopus microsporus	Germany
NRZ-2018-419	Rhizopus arrhizus	Germany
NRZ-2018-423	Rhizopus microsporus	Germany
NRZ-2018-463	Rhizopus arrhizus	Germany
NRZ-2018-475	Rhizopus arrhizus	Germany
NRZ-2018-476	Rhizopus arrhizus	Germany
NRZ-2018-478	Rhizopus arrhizus	Germany
NRZ-2018-560	Rhizopus arrhizus	Germany
NRZ-2018-581	Rhizopus microsporus	Germany
NRZ-2018-591	Rhizopus arrhizus	Germany
S1433	*Mortierella polygonia*	Antarctica
S1bC	*Mortierella elongata*	Iceland
S1bD	*Mortierella elongata*	Iceland
S2bC	*Mortierella elongata*	Iceland
S3123A	*Mortierella alpina*	Antarctica
S3323	*Mortierella alpina*	Antarctica
S3421	*Mortierella alpina*	Antarctica
MGC163b	*Umbelopsis* sp.	Poland
MGC164	*Umbelopsis* sp.	Poland
U25	*Umbelopsis* sp.	Poland
U34	*Umbelopsis angularis*	Poland
U41	*Umbelopsis angularis*	Poland
U810	*Umbelopsis angularis*	Poland
WA18942	*Mortierella* sp.	Poland
WA18944	*Mortierella calciphila*	Poland
WA49853	*Mortierella formicae*	Poland
WA50677	*Mortierella verticillata*	Poland
WA50678	*Mortierella* sp.	Poland
WA50678	*Mortierella elongata*	Poland
GRM41	*Mortierella elongata*	Poland
WA50679	*Mortierella rishikesha*	Poland
WA50680	*Mortierella elongata*	Poland
WA50680	*Mortierella elongata*	Poland
WA50681	*Mortierella elongata*	Poland
WA50682	*Umbelopsis ramanniana sensu lato*	Poland
WA50683	*Mortierella zychae*	Poland
WA50684	*Mortierella zychae*	Poland
WA50685	*Mortierella elongata*	Poland
WA50687	*Mortierella elongata*	Poland
WA50688	*Mortierella elongata*	Poland
WA50689	*Mortierella elongata*	Poland
WA50691	*Mortierella elongata*	Poland
WA50692	*Umbelopsis ramanniana sensu lato*	Poland
WA50693	*Umbelopsis ramanniana sensu lato*	Poland
WA50694	*Umbelopsis ramanniana sensu lato*	Poland
WA50697	*Umbelopsis ramanniana sensu lato*	Poland
WA50698	*Umbelopsis ramanniana sensu lato*	Poland
WA50699	*Umbelopsis ramanniana sensu lato*	Poland
WA50700	*Umbelopsis ramanniana sensu lato*	Poland
WA50701	*Umbelopsis ramanniana sensu lato*	Poland
WA50702	*Umbelopsis ramanniana sensu lato*	Poland
WA50703	*Umbelopsis isabellina*	Poland
WA50704	*Umbelopsis ramanniana sensu lato*	Poland
WA50705	*Umbelopsis ramanniana sensu lato*	Poland
WA50706	*Umbelopsis ramanniana sensu lato*	Poland
WA50707	*Umbelopsis ramanniana*	Poland
WA50719	*Umbelopsis ramanniana sensu lato*	Poland
WA50720	*Umbelopsis ramanniana sensu lato*	Poland
WA51536	*Umbelopsis vinacea*	Poland
WA67140	*Mortierella gamsii*	Romania
WA67141	*Mortierella alpina*	Romania
WA67145	*Mortierella zychae*	Poland
WA67154	Rhizopus microsporus	Poland
WA67162	*Mortierella gamsii*	Romania
WA67163	*Mortierella hyalina*	Poland
WA67166	*Mortierella* sp.	Poland
WA67171	*Mortierella alpina*	Poland
WA67176	*Mortierella* sp.	Chile
WA67179	*Mortierella gemmifera*	Chile
WA67203	*Mortierella zychae*	Poland
WA67204	*Mortierella zychae*	Poland
WA67205	*Mortierella* sp.	Poland
WA67206	*Mortierella zychae*	Poland
WA67211	*Mortierella zychae*	Poland
WA67219	*Mortierella* sp.	Chile
WA71869	*Umbelopsis ramanniana sensu lato*	Poland
WA71874	*Umbelopsis* sp.	Poland
WA71875	*Umbelopsis isabellina*	Poland
WA71876	*Umbelopsis ramanniana sensu lato*	Poland
WA71877	*Mortierella* sp.	Poland
WA71878	*Umbelopsis ramanniana*	Poland
WA71879	*Umbelopsis ramanniana*	Poland
WA71880	*Umbelopsis ramanniana*	Poland
WA71881	*Umbelopsis ramanniana*	Poland
WA71883	*Umbelopsis ramanniana*	Poland
WA71884	*Umbelopsis ramanniana*	Poland
WA71885	*Umbelopsis ramanniana*	Poland
WA71887	*Umbelopsis ramanniana*	Poland
WA71891	*Umbelopsis angularis*	Poland
WA71892	*Umbelopsis vinacea*	Poland
WA74572	*Umbelopsis* sp.	Poland
WA74573	*Umbelopsis* sp.	Poland
XWhI	*Mortierella bainieri*	The Arctic
XWhII	*Mortierella minutissima*	The Arctic
Zl7	*Claroideoglomus claroideum*	Poland
ZR16	*Diversispora* sp.	Poland
ZR16 Se-3	*Diversispora* sp.	Poland
GMR42	*Mortierella elongata*	Poland
GRM50A	*Mortierella elongata*	Poland
GRM51prim	*Mortierella elongata*	Poland
GRM51A	*Mortierella elongata*	Poland

a ND, no data.

### DNA extraction, amplification, and sequencing.

Whole genomic DNA was extracted using ExtractMe genomic DNA kit (Blirt S.A., Gdańsk, Poland) according to the manufacturer’s protocol. An internal transcribed spacer (ITS) rRNA gene fragment was amplified using a 20-μl PCR mixture, which consisted of 10 μl of 2× TaqNova-RED PCR master mix (Blirt S.A., Gdańsk, Poland), 1.5 μl each of ITS1f and ITS4 primers in 10 pmol μl^−1^ concentration (35), up to 7 μl of template DNA (depending on the template’s concentration), and distilled water up to 20 μl. PCR was performed as follows: 4 min in 95°C for initial denaturation, 35 cycles of 30 s in 95°C, 30 s in 54°C, 1 min in 72°C for annealing, and 10 min in 72°C for final elongation.

PCR amplicons were visualized by 1% agarose gel electrophoresis and purified using ExtractMe genomic clean up kit (Blirt S.A., Gdańsk, Poland) and used as a template for Sanger sequencing with the ABI PRISM BigDye Terminator cycle sequencing ready reaction kit 3.1 (Applied Biosystems, Warrington, UK) with the same primers as those used in PCR. Sequencing was outsourced to Genomed (Genomed S.A., Warszawa, Poland). A BLASTn search (36) against the UNITE database (https://unite.ut.ee) (37) was performed for the obtained ITS fragments in order to assess taxonomic placement of each strain. Fungal sequence data generated for this study are available in GenBank under accession numbers MT009408 to MT009438 and MT009444 to MT009481.

### Detection of endofungal bacteria.

DNA isolates from each strain were then used as a template for PCR targeting bacterial 16S rRNA genes. PCR was performed in a 25-μl volume consisting of 2.5 μl of 10× DreamTaq green buffer (Thermo Fisher Scientific, Waltham, MA, USA), 0.5 μl of deoxynucleoside triphosphates (dNTPs) mix (Thermo Fisher Scientific, Waltham, MA, USA), 0.5 μl of each of two universal bacterial primers, i.e., 27F (5′-AGAGTTTGATCCTGGCTCAG-3′) and 1492R (5′-GGTTACCTTGTTACGACTT-3′) in 10 pmol μl^−1^ concentration, 0.1 μl *Taq* DNA polymerase (Qiagen, Hilden, Germany), 1 μl of template DNA, and 19.9 μl of distilled water. For difficult templates (resulting in a small amount of expected product), PCR was repeated using *Taq* PCR core kit (Qiagen, Hilden, Germany) using the same template as before. Reaction was performed in a 50-μl volume consisting of 5 μl of 10× CoralLoad PCR buffer, 10 μl of 5× Q-solution, 1 μl of dNTPs mix (Thermo Fisher Scientific, Waltham, MA, USA), 2.5 μl of each primer, 0.25 μl of *Taq* DNA polymerase, at least 3 μl of template DNA (depending on concentration), and distilled water up to 50 μl. PCR was performed as follows: 3 min in 94°C for initial denaturation, 35 cycles of 30 s in 94°C, 30 s in 53°C, 1 min in 72°C for annealing, and 10 min in 72°C for final elongation. Presence of the PCR product was confirmed on 1% agarose gel and then purified and cloned.

### Cloning of 16S rRNA gene PCR products.

Purified 16S rRNA genes were then cloned on pGEM-T Easy vector using pGem-T Easy vector systems kit (Promega Corporation, Madison, WI, USA) according to the manufacturer’s protocol and transformed into Dh5-alpha-competent E. coli cells (MCLAB, San Francisco, CA, USA). Transformed Dh5-alpha cells were plated on LB agar medium (Sigma-Aldrich, Saint Louis, MO, USA) supplemented with 200 μl of 5-bromo-4-chloro-3-indolyl-β-d-galactopyranoside (X-gal), 200 μl of isopropyl-β-d-thiogalactopyranoside (IPTG), and 200 μl of ampicillin per 100 ml of medium. Transformants of each strain were plated twice, and successfully cloned colonies were chosen from each plate for further investigation.

Subsequently, direct PCR was performed for each colony in a 20-μl volume consisting of 10 μl 2× TaqNova-RED PCR master mix (Blirt S.A., Gdańsk, Poland), 1.5 μl (each) of M13F and M13R primers, 7 μl of distilled water, and a small amount of material from the bacterial colony. PCR was performed as follows: 3 min in 95°C for initial denaturation, 35 cycles of 30 s in 95°C, 30 s in 55°C, 1 min in 72°C for annealing, and 5 min in 72°C for a final elongation. Presence of the PCR product was then confirmed on 1% agarose gel and purified as described previously. Five DNA clones from each fungal strain were used as a template for Sanger sequencing and sequenced as described previously. Bacterial sequence data generated for this study are available in GenBank under accession numbers MT002691 to MT002716, MW055707 to MW055867, and MW080027 to MW080031 (BRE) and MT031989 to MT032002 (MRE).

### Phylogenetic analyses of detected endobacteria.

Forward and reverse reads of 16S rRNA gene sequences obtained in the previous step were assembled using Geneious Prime 2019.2 (Geneious, Auckland, New Zealand). Sequences belonging to the *Burkholderiaceae* family were selected using BLASTn searches (36) against the NCBI database (38). Only these sequences were used for further analysis. Then, if the sequences assembled from clone reads from one fungal strain were similar in at least 98%, a consensus sequence from them was created using cons 6.6.0.0 from the EMBOSS package (39). If not, the sequences were used separately. We then combined obtained sequences with publicly available 16S rRNA gene sequences of previously detected BRE and free-living *Burkholderiaceae*, aligned them together using MAFFT (v.7.271; –auto) (40), and trimmed them using trimAl (v.1.2rev59; -automated1) (41). Trimmed alignment was then visually inspected and used for calculating a phylogenetic tree. ModelTest-NG was used to check which evolutionary model of substitutions should be used (TIM3 + I + G4), and RAxML-NG (v.0.8.0) was subsequently used for finding the best tree and calculating 1,000-bootstrap replicates (42).

The same alignment was used for finding the best Bayesian tree using MrBayes (43, 44) with the best fit model of nucleotide evolution (generalized time reversible [GTR] and inverted gamma-distributed rate variation). Metropolis-coupled Markov chain Monte Carlo (MCMC) chains were run for 500,000 generations, with trees sampled every 100 generations, and an initial burn-in threshold was set to 1,250 trees; from the remaining trees, the consensus phylogram was computed using the 50% majority rule.

To establish whether endohyphal bacteria coevolved with their fungal hosts, we used the global fits method. First, sequences of small and large ribosomal subunits for each fungal host were obtained from NCBI (accession numbers used for the tree can be found in Table S2 in the supplemental material), aligned separately using MAFFT, trimmed using trimAl, and, after separately checking for the best evolutionary model using ModelTest-NG, concatenated. Then, the fungal tree was calculated using RAxML-NG with the same settings as for the bacterial tree described above. We also calculated two separate phylogenetic trees for bacteria, one for *Paraburkholderia* sequences and one for *Burkholderia sensu lato*, using the same software and settings as before. Afterward, the global hypothesis of coevolution between fungal hosts and harbored bacteria was tested for both groups using the ParaFit function from ape v.5.3 R package (45) with 999 permutations to implement a global test as well as individual links. The interaction was considered to be significant if the ParaFitGlobal *P* value was <0.05. Individual links between hosts and bacteria were visualized on tanglegrams (Fig. 2) created using phytools v.0.6.99 R package (46).

All of the trees and tanglegrams were edited using FigTree (47), iTOL (48), and Inkscape (49) software.

### Visualization of endobacteria.

The strains from different orders of Mucoromycota (namely, Mortierellales, Umbelopsidales, and Mucorales; strains *Mortierella elongata* WA50687, *Umbelopsis* sp. WA50699, Rhizopus microsporus WA67154) putatively harboring EHB were chosen for visualization procedure. Small (0.5 cm^2^) fragments of fungal cultures were taken from 2% MEA plates, washed in 1× phosphate-buffered saline (PBS) three times, and fixed in 10% formalin (no additional permeabilization was implemented). Samples were then centrifuged at 4,500 rpm for 8 min and incubated for 3 h at 4°C. Subsequently, samples were centrifuged with 4,500 rpm for 8 min, after which supernatant was replaced with autoclaved Milli-Q water. This last procedure was repeated twice, and after the last round of centrifuging, biomass was suspended in 1× PBS (pH 7.4). All samples were stored at −20°C until further analyses were performed.

Fluorescence *in situ* hybridization (FISH) analyses were performed according to Nielsen et al. (50) with the following modifications. The procedure was performed in suspension instead of slides. At least 2 μg of each fungal colony was suspended in 40 μl of hybridization buffer and incubated at 46°C overnight. Then, samples were centrifuged, and hybridization buffer was replaced by washing buffer. After 15 min of washing at 48°C, washing buffer was discarded, and samples were resuspended in cold distilled water (dH_2_O). Finally, samples were transferred to wells on slides in proper aliquot to obtain a thin hyphal layer, facilitating microscopic observation (volumes between 5 and 40 μl were tested). Fungal biomass after FISH procedure without addition of probe was used as the negative control. The negative control was needed to assess autofluorescence.

The fungal hyphae were recognized by bright-field microscopy; then, the endohyphal bacteria were stained by FISH universal bacterial probe EUB338 labeled with Cy3 and observed under a microscope with a proper set of filters (excitation, 552 nm; emission, 565 nm). Detailed probe information is available in probeBase (51). The EHB were visualized using an Olympus IX81F– ZDC2 confocal microscope and Andor iQ software, objectives CLARA100×/60×/40×.

### Data availability.

Sequences produced in the study can be found in the NCBI database under GenBank accession numbers MT031989 to MT032002 (MRE), MT009408 to MT009438 and MT009444 to MT009481 (fungal ITS), MT002691 to MT002716, MW055707 to MW055867, and MW080027 to MW080031 (BRE).

## Supplementary Material

Supplemental file 1

Supplemental file 2
